# Systemic Cytokine Patterns and Histologic Disease Spectrum in Inflammatory Bowel Disease

**DOI:** 10.3390/cimb48050516

**Published:** 2026-05-15

**Authors:** Nikolaos Martinos, Christos Kroupis, Maria Gypari, Georgios Kranidiotis, Christos Karakoidas, Marina Konstantinou, Andreas C. Lazaris, Georgia-Eleni Thomopoulou

**Affiliations:** 1 Gastroenterology Department, Naval Hospital of Athens, 115 21 Athens, Greece; g.kranidiotis@gmail.com (G.K.); chkarako@gmail.com (C.K.); 2 Department of Clinical Biochemistry, Attikon University General Hospital, National and Kapodistrian University of Athens, 124 62 Athens, Greece; ckroupis@med.uoa.gr; 3 Department of Pathology, Naval Hospital of Athens, 115 21 Athens, Greece; mgypari1505@gmail.com (M.G.); marinakonst@yahoo.gr (M.K.); 4 First Department of Pathology, School of Medicine, National and Kapodistrian University of Athens, 115 27 Athens, Greece; alazaris@med.uoa.gr; 5 Cytopathology Department, Attikon University General Hospital, School of Medicine, National and Kapodistrian University of Athens, 124 62 Athens, Greece; gthomop@med.uoa.gr

**Keywords:** inflammatory bowel disease, histologic mucosal healing, Geboes score, interleukin-10, interleukin-23, cytokine signaling

## Abstract

Background/Objectives: Histologic mucosal healing is an increasingly recognized therapeutic target in inflammatory bowel disease (IBD), yet reliable non-invasive correlates remain limited. This study aimed to evaluate circulating cytokine patterns as detectability-based immune signals across the spectrum of histologic disease activity. Methods: In this prospective cross-sectional study, 59 patients with IBD and 36 healthy controls were enrolled. Serum interleukin-10 (IL-10) and interleukin-23 (IL-23) were quantified by ELISA. Histologic activity was graded using the Geboes score. Associations were assessed using non-parametric methods and multivariable logistic regression with Firth penalization. Results: IL-10 demonstrated apparent separation across histologic states, primarily driven by reduced detectability in active inflammation, and was inversely associated with histologic severity. IL-10 remained associated with histologic status, although estimates should be interpreted cautiously. Detectable IL-23 was confined to moderate-to-severe inflammation and did not show graded discrimination, with interpretation limited by the small number of detectable observations. Conclusions: IL-10 and IL-23 exhibit complementary patterns, reflecting detectability-based regulatory signaling and a severity-dependent inflammatory threshold, respectively, without evidence of independent clinical utility for IL-23 in the present dataset. These findings are exploratory and require validation in larger prospective cohorts.

## 1. Introduction

Inflammatory bowel disease (IBD), including ulcerative colitis (UC) and Crohn’s disease (CD), is characterized by chronic relapsing intestinal inflammation driven by dysregulated immune responses. Although therapeutic strategies have traditionally focused on symptomatic control and endoscopic remission, accumulating evidence indicates that persistent microscopic inflammation may remain despite apparent mucosal healing and is associated with adverse clinical outcomes, including relapse, corticosteroid exposure, hospitalization, and disease progression [1,2]. Consequently, histologic mucosal healing has emerged as a more stringent therapeutic target reflecting deeper disease control.

Histologic assessment requires invasive endoscopy with biopsy sampling and expert pathological interpretation, limiting its feasibility for repeated monitoring in routine clinical practice. Conventional non-invasive biomarkers, including C-reactive protein (CRP) and fecal calprotectin (FC), provide indirect estimates of inflammatory burden but may not consistently distinguish histologic healing from residual microscopic inflammation [3,4]. This limitation has increased interest in systemic immune biomarkers capable of reflecting inflammatory and regulatory pathways more directly.

The IL-23/Th17 signaling axis plays a central role in intestinal inflammation through maintenance of pathogenic effector responses and amplification of pro-inflammatory cytokine networks [5,6,7,8,9,10,11]. Therapeutic targeting of IL-23 pathways has demonstrated clinical efficacy in both UC and CD, further supporting its biological relevance in IBD pathogenesis [12,13,14].

In contrast, IL-10 is a key immunoregulatory cytokine involved in suppression of antigen-presenting cell activation and maintenance of intestinal immune tolerance [15,16,17,18]. Defects in IL-10 signaling have been linked to severe intestinal inflammatory phenotypes, emphasizing its importance in mucosal immune homeostasis.

Based on the complementary immunologic roles of IL-10 and IL-23, we hypothesized that circulating cytokine patterns may associate with histologic disease activity in IBD. Therefore, the present study evaluated serum IL-10 and IL-23 in relation to histologic status as assessed by the Geboes score in a prospective clinical cohort, with emphasis on detectability-based cytokine behavior and exploratory association with microscopic inflammation.

## 2. Materials and Methods

### 2.1. Study Design and Participants

This was a prospective, single-center, observational cross-sectional study conducted at the Hepato-Gastroenterology Unit of the Naval Hospital of Athens, Greece. Screening of potentially eligible patients began in March 2025 among individuals scheduled to undergo clinically indicated colonoscopy. However, all patient enrollment, informed consent, and study-related procedures were conducted only after ethics approval was obtained in April 2025, with recruitment continuing through August 2025.

The source population consisted of adult patients with established inflammatory bowel disease (IBD) undergoing routine clinical evaluation during this period. All eligible patients meeting predefined inclusion and exclusion criteria were invited to participate following ethics approval.

Eligible participants included adult patients (≥18 years) with a confirmed diagnosis of IBD, including ulcerative colitis and Crohn’s disease, established on the basis of clinical, endoscopic, radiologic, and histopathologic criteria according to international guidelines [19,20,21,22]. Patients were eligible irrespective of disease duration, clinical disease stage, or prior treatment exposure, provided that they were clinically stable and able to undergo colonoscopy at the time of enrollment. Clinically stable was defined as absence of acute disease flare, hospitalization, or escalation of systemic corticosteroid therapy within the preceding four weeks.

Ongoing maintenance therapies, including 5-aminosalicylic acid (5-ASA), biologic therapy, and combination regimens, were recorded and incorporated into the analytical plan as covariates to account for potential immunomodulatory effects.

A control group consisted of individuals undergoing screening colonoscopy for preventive purposes, without known inflammatory, autoimmune, or malignant disease and with macroscopically and histologically normal colonic mucosa.

Exclusion criteria included pregnancy, active infection, known malignancy, severe organ failure, antibiotic use within four weeks prior to enrollment, and prior exposure to monoclonal antibodies targeting the IL-23 pathway within the preceding 12 months. Individuals with significant uncontrolled systemic comorbidities that could influence inflammatory biomarker levels were also excluded.

Participants were enrolled consecutively to reduce the risk of selection bias. The study was conducted and reported in accordance with the Strengthening the Reporting of Observational Studies in Epidemiology (STROBE) guidelines. No missing data were observed for primary variables (histology, IL-10, IL-23, and CRP), reflecting a complete-case dataset achieved through prospective data collection and predefined data quality control procedures.

### 2.2. Ethical Approval

The study was conducted in accordance with the Declaration of Helsinki (1975, revised in 2013) and approved by the Institutional Review Board of the Naval Hospital of Athens (protocol code 3366, approved on 11 April 2025) and by the Institutional Review Board of Attikon University Hospital (protocol code 219, approved on 8 April 2025). Written informed consent was obtained from all participants prior to enrollment. All participant data were handled in accordance with applicable data protection and confidentiality regulations. Personal identifiers were removed prior to analysis, and data were anonymized to ensure participant privacy.

### 2.3. Blood Sampling and Processing

Peripheral venous blood samples were obtained immediately prior to colonoscopy and before any endoscopic manipulation. Serum was separated by centrifugation within two hours of collection and stored at −80 °C until analysis.

Samples were stored for comparable durations across study groups. The first collected sample was analyzed approximately six months after collection, and the overall median storage duration was approximately three months. All samples were analyzed in batch during the same study period to minimize inter-assay variability. Each sample underwent a single freeze–thaw cycle prior to cytokine quantification, a procedure considered acceptable for minimizing protein degradation based on prior methodological studies. Although storage durations were comparable across study groups, the potential impact of storage time on cytokine stability cannot be fully excluded and should be considered when interpreting the results.

### 2.4. Cytokine Quantification

Serum interleukin-10 (IL-10) and interleukin-23 (IL-23) concentrations were measured using commercially available sandwich enzyme-linked immunosorbent assay (ELISA) kits (Human IL-10 Quantikine ELISA Kit, catalog no. D1000B; Human IL-23 Quantikine ELISA Kit, catalog no. D2300B; R&D Systems, Minneapolis, MN, USA) according to the manufacturer’s instructions.

All samples and standards were measured in duplicate, and mean values were used for statistical analysis. Optical density was measured at 450 nm with wavelength correction using a Bio-Tek ELx800 microplate reader (BioTek Instruments, Winooski, VT, USA). Cytokine concentrations were calculated using four-parameter logistic (4PL) curve fitting with GEN5 software (version 3.11). Calibration curves were generated for each assay plate using manufacturer-provided standards and applied to sample quantification within the same plate to ensure consistency of measurement across plates.

The lower limits of detection (LOD) were 3.9 pg/mL for IL-10 and 16.3 pg/mL for IL-23. The dynamic quantifiable ranges were 7.8–500 pg/mL for IL-10 and 39–2500 pg/mL for IL-23.

Observed assay precision in our laboratory demonstrated intra-assay coefficients of variation (CVs) ranging from 5.3% to 7.2% and inter-assay CVs ranging from 8.2% to 10.1% for IL-10, and intra-assay CVs ranging from 5.5% to 7.8% and inter-assay CVs ranging from 7.1% to 10.5% for IL-23. These values were consistent with manufacturer-reported performance characteristics.

All cytokine measurements were performed in batch during the same study period, and laboratory personnel were blinded to clinical, endoscopic, and histologic data, minimizing the risk of measurement bias.

### 2.5. Handling of Detection Limits

Given the presence of left-censored cytokine measurements below the assay limit of detection (LOD), prespecified analytical strategies were applied with emphasis on minimizing overinterpretation of values below the detection threshold.

For IL-10, a substantial proportion of measurements fell below the LOD (3.9 pg/mL), particularly among patients with active histologic inflammation. Accordingly, IL-10 was analyzed primarily as a detectability-based variable (detectable ≥3.9 pg/mL vs. non-detectable <3.9 pg/mL), providing a clinically interpretable representation of the biomarker signal that does not rely on distributional assumptions.

For descriptive and exploratory purposes, values below the LOD were additionally imputed as LOD/2 (1.95 pg/mL), and IL-10 was examined as a log-transformed continuous variable. However, given the high proportion of non-detectable values, continuous analyses were interpreted cautiously and considered supportive rather than primary.

For IL-23, given the very high proportion of non-detectable values, analyses were based primarily on detectability status (non-detectable <16.3 pg/mL vs. detectable ≥16.3 pg/mL). Sensitivity analyses further examined ordinal categorization (<LOD; 16.3–38.9 pg/mL; ≥39 pg/mL) to account for the lower limit of quantification (LLOQ).

The use of LOD/2 imputation represents a pragmatic approach commonly applied in exploratory biomarker studies to retain censored observations; however, in the present study, results derived from continuous modeling are interpreted with caution due to the high degree of left-censoring. Detectability-based analyses were therefore prioritized for primary interpretation.

This approach avoids treating imputed LOD values as precise quantitative measurements in groups with near-complete non-detectability.

In the present dataset, IL-10 values were below the detection limit in 67.8% of IBD patients overall (97.1% in active histologic inflammation), whereas IL-23 values were below detection in 89.8% of the IBD cohort. These distributions informed the analytical strategy emphasizing detectability-based interpretation.

### 2.6. Endoscopic and Histologic Assessment

Colonoscopy was performed according to standard clinical practice. Bowel preparation quality was assessed using the Boston Bowel Preparation Scale (BBPS), and only procedures with adequate preparation were included in the analysis.

Mucosal biopsies were obtained during colonoscopy for histologic evaluation. In the control group, biopsies were collected from endoscopically normal-appearing colonic mucosa. In patients with inflammatory bowel disease, biopsies were obtained either from endoscopically inflamed mucosa or from macroscopically normal mucosa when no visible inflammatory lesions were present.

Endoscopic activity in ulcerative colitis was assessed using the Mayo endoscopic subscore, and in Crohn’s disease using the Simple Endoscopic Score for Crohn’s Disease (SES-CD).

Histologic evaluation was performed using the Geboes scoring system by two experienced gastrointestinal pathologists who were blinded to clinical, endoscopic, and cytokine data. Discrepancies were resolved by consensus.

Histologic activity was graded using the Geboes score (range 0–5.4), which evaluates architectural changes and inflammatory features, including chronic inflammatory infiltrate, lamina propria neutrophils and eosinophils, cryptitis, crypt abscesses, and epithelial damage, erosion, or ulceration. Histologic mucosal healing was defined a priori as a Geboes score < 2.0.

### 2.7. Treatment Classification

At the time of colonoscopy, patients were categorized according to ongoing maintenance therapy into four groups: no treatment, treatment with 5-aminosalicylic acid (5-ASA), advanced biologic therapy, or combination therapy consisting of a biologic agent plus azathioprine. No patients were receiving monoclonal antibodies targeting the IL-23 pathway at the time of enrollment.

### 2.8. Laboratory Measurements

C-reactive protein (CRP) was measured using standard automated laboratory methods at the hospital’s accredited clinical laboratory.

### 2.9. Outcomes

The primary analytical framework of the study focused on histologic disease status within the IBD cohort, while also examining cytokine patterns across the broader spectrum of disease states, including healthy controls.

Within the IBD cohort, the primary comparison evaluated cytokine levels according to histologic mucosal healing (Geboes score < 2) versus histologic activity (Geboes score ≥ 2). In parallel, analyses were performed using continuous histologic severity (Geboes score) to assess associations across the full spectrum of microscopic inflammation.

The healthy control group was included as an external reference to characterize baseline circulating cytokine levels and to contextualize cytokine patterns across health, histologic healing, and active disease. Controls were not included in primary within-cohort analyses but were incorporated in descriptive and comparative analyses across disease states.

Secondary outcomes included the association between cytokine levels or detectability and histologic status, the correlation of cytokine levels with continuous histologic severity, and the evaluation of independent associations in multivariable models. Additional exploratory analyses assessed cytokine behavior using alternative modeling approaches (continuous versus detectability-based) and according to treatment exposure.

Comparisons between ulcerative colitis and Crohn’s disease were performed as secondary exploratory analyses to assess potential disease-specific patterns.

### 2.10. Statistical Analysis

All analyses were conducted using R version 4.4.0 (R Foundation for Statistical Computing, Vienna, Austria). Continuous variables are presented as median (interquartile range), and categorical variables as number (percentage). All tests were two-sided, and *p*-values < 0.05 were considered statistically significant.

The primary analytical approach focused on describing cytokine patterns across histologic disease states and evaluating associations with both binary histologic status and continuous histologic severity.

Between-group comparisons of IL-10 concentrations were performed using Kruskal–Wallis tests followed by pairwise Mann–Whitney U tests with Holm correction. Associations with continuous Geboes scores were assessed using Spearman’s rank correlation. Group comparisons for IL-23 detectability were performed using Fisher’s exact test or Pearson’s chi-square test as appropriate, with Monte Carlo simulation applied for sparse contingency tables.

Multivariable logistic regression was used to evaluate independent associations with histologic mucosal healing. Given the limited number of outcome events and potential quasi-complete separation, penalized logistic regression using Firth’s correction (logistf package) was prespecified to mitigate small-sample bias. IL-10 was entered as a log-transformed continuous variable, and IL-23 as detectable versus non-detectable. Covariates were prespecified based on biological plausibility and clinical relevance (age, sex, CRP, treatment category), and no data-driven variable selection procedures were performed.

Receiver operating characteristic (ROC) curves were used as a secondary descriptive measure of discrimination. Areas under the curve (AUCs) with 95% confidence intervals were calculated using DeLong’s method, and optimal cut-off values were estimated using the Youden index. Internal validation was performed using bootstrap resampling (2000 iterations) to assess model stability and estimate optimism-corrected performance. Calibration was evaluated using calibration slope and intercept derived from logistic regression of observed outcomes on predicted probabilities, and overall predictive accuracy was quantified using the Brier score.

For ROC analyses, IL-23 was modeled using non-detectability as the healing-favoring direction to reflect the absence of circulating IL-23 in histologic remission, whereas detectability (≥16.3 pg/mL) was retained in regression models to preserve biological interpretability.

### 2.11. Sample Size Justification

The final sample comprised 59 patients with IBD, including 24 (40.7%) with histologic mucosal healing. Given the exploratory nature of the study and the absence of prior effect size estimates for circulating IL-10 in relation to histologic remission, a formal a priori sample size calculation was not feasible.

With 24 healing events and 8 parameters included in the multivariable model, the events-per-variable (EPV) ratio was 3.0. To address potential small-sample bias and quasi-complete separation, penalized logistic regression using Firth’s correction was prespecified.

For discrimination analysis, assuming a null AUC of 0.70 and an observed AUC of 0.871 for continuous IL-10, the available sample corresponds to a large apparent discrimination effect within this dataset. However, given the limited sample size and exploratory design, these estimates should be interpreted descriptively rather than as precise measures of performance.

Accordingly, the study was sufficiently powered to detect moderate-to-large effects within this cohort but should be considered hypothesis-generating. External validation in independent cohorts is required to assess generalizability.

### 2.12. Data Availability

The dataset generated and analyzed during the current study is available from the corresponding author upon reasonable request.

## 3. Results

### 3.1. Study Population and Histologic Classification

Potentially eligible participants were identified between March and August 2025 at the Hepato-Gastroenterology Unit of the Naval Hospital of Athens. Formal recruitment, informed consent, and all study-related procedures were conducted consecutively between April and August 2025, following institutional IRB approval.

**Figure 1:**
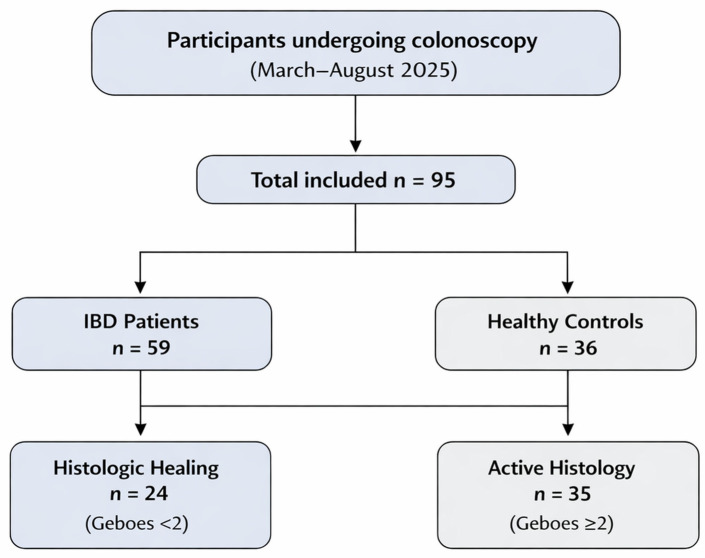
Study participant flow diagram. Participants undergoing colonoscopy at the Hepato-Gastroenterology Unit of the Naval Hospital of Athens between March and August 2025 were consecutively recruited and assessed for eligibility. A total of 95 participants were included in the final analysis, comprising 59 patients with inflammatory bowel disease (IBD) and 36 healthy controls. Within the IBD cohort, 24 patients demonstrated histologic mucosal healing (Geboes < 2), while 35 had active histologic inflammation (Geboes ≥ 2).

The participant recruitment and inclusion process is illustrated in Figure 1.

A total of 95 individuals were included, comprising 59 patients with IBD and 36 healthy controls. Among patients with IBD, 24 (40.7%) achieved histologic mucosal healing (Geboes < 2), while 35 (59.3%) had active microscopic inflammation.

Baseline demographic characteristics were comparable across study groups, including healthy controls and IBD subgroups. Age (*p* = 0.18) and sex distribution (*p* = 0.21) did not differ significantly, indicating no evidence of demographic imbalance. In contrast, CRP levels differed significantly across groups, with higher values observed in patients with active histologic inflammation (*p* < 0.001). These data are summarized in Table 1.

### 3.2. Endoscopic–Histologic Relationship

Endoscopic scores differed significantly according to histologic classification. In ulcerative colitis, the Mayo endoscopic subscore was lower in the histologic healing group (*p* = 0.0007). In Crohn’s disease, SES-CD scores were also significantly lower among patients with histologic healing (*p* = 0.0072).

Despite this association, histologic inflammation was observed in a subset of patients with low endoscopic scores, indicating incomplete concordance between endoscopic and microscopic disease activity (Table 2).

### 3.3. Treatment Distribution

Treatment exposure at the time of colonoscopy showed a borderline association with histologic status (Fisher’s exact test with Monte Carlo simulation, *p* = 0.0549; Cramér’s V = 0.359). Untreated patients were observed exclusively in the active histology group, whereas rates of biologic therapy were comparable between groups (Table 3).

### 3.4. Serum IL-10 and Histologic Activity

**Figure 2:**
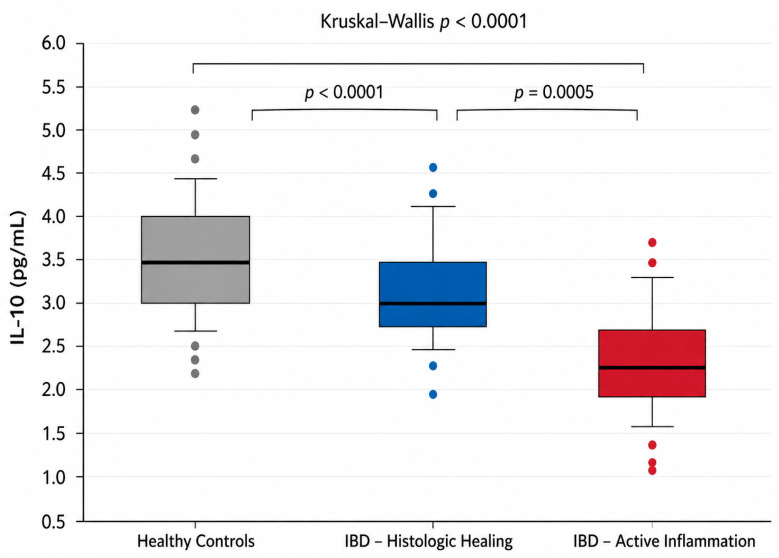
Serum IL-10 levels are shown for healthy controls, IBD patients with histologic mucosal healing (Geboes < 2), and IBD patients with active histologic inflammation. Boxes represent the interquartile range (IQR), horizontal lines indicate the median, and whiskers denote the distribution according to the boxplot method. Overall differences across groups were assessed using the Kruskal–Wallis test (*p* < 0.0001), followed by pairwise Mann–Whitney U tests with Holm correction.

Serum IL-10 concentrations showed apparent separation across disease states, primarily driven by clustering at the detection threshold in active disease (Figure 2).

Median (interquartile range) IL-10 concentrations showed marked separation across groups, with higher values in healthy controls (12.1 [8.8–18.7] pg/mL), intermediate values in IBD patients with histologic mucosal healing (4.5 [3.5–5.4] pg/mL), and values clustered at the assay detection threshold in patients with active histology (1.95 [1.95–1.95] pg/mL) (Kruskal–Wallis H = 81.454, *p* < 0.0001; Table 4).

When examined across disease states, patients with IBD exhibited markedly lower circulating IL-10 concentrations compared with healthy controls (median 3.3 pg/mL [IQR 2.5–4.3] vs. 12.1 pg/mL [8.8–18.7]; Mann–Whitney U test, *p* < 0.0001), reflecting a progressive loss of IL-10 detectability from health to intestinal inflammation rather than a fully continuous quantitative gradient.

A substantial proportion of IL-10 measurements fell below the assay limit of detection. IL-10 values were below the lower limit of detection (3.9 pg/mL) in 67.8% of IBD patients overall, including 97.1% of patients with active histologic inflammation compared with 25% of those with histologic healing. The uniform values in the active group reflect clustering at the detection threshold rather than measurable biological variability, consistent with near-complete non-detectability in this subgroup.

Accordingly, IL-10 was interpreted primarily as a detectability-based marker, with detectable IL-10 predominantly observed in patients with histologic healing and non-detectable IL-10 strongly enriched among patients with active microscopic inflammation.

Continuous analyses of IL-10 were therefore considered exploratory and supportive, given the high proportion of left-censored values. Within this context, IL-10 concentrations showed a directional inverse association with histologic severity, primarily driven by detectability differences (Spearman ρ = −0.766, *p* = 1.61 × 10^−12^), reflecting directional association rather than a precise dose–response relationship.

In regression analyses, higher IL-10 levels (log-transformed, LOD-adjusted) were associated with histologic status. After multivariable Firth penalized adjustment for age, sex, CRP, IL-23 detectability, and treatment category, IL-10 remained independently associated with histologic mucosal healing (adjusted OR 47.25; 95% CI 5.79–385.46; *p* = 0.00032) (Table 5). The large effect estimates and wide confidence intervals reflect near-complete separation driven by the high prevalence of non-detectable IL-10 in active disease and should be interpreted as indicative of a strong directional signal rather than a precise quantitative effect estimate.

The consistency between detectability-based and continuous exploratory analyses supports the presence of a robust association, although this should not be interpreted as evidence of precise quantitative discrimination in the setting of high LOD-related clustering.

In analyses stratified by treatment exposure, circulating IL-10 concentrations did not differ significantly between patients receiving biologic therapy (advanced or combination regimens) and those receiving non-biologic treatment (Mann–Whitney U test, *p* = 0.54).

In sensitivity analyses excluding untreated patients, the association between IL-10 concentrations and histologic status remained consistent (*p* < 0.001), further supporting the robustness of the detectability-driven pattern across treatment strata.

### 3.5. Sensitivity Analysis: IL-10 Detectability

Given the high proportion of values below the assay limit of detection, IL-10 was interpreted primarily as a detectability-based biomarker, independent of continuous modeling and LOD-based imputation.

Detectable IL-10 (≥3.9 pg/mL) was present in 18/24 patients (75.0%) with histologic healing and in only 1/35 patients (2.9%) with active histology, demonstrating near-complete separation between histologic groups based on detectability status.

In univariable analysis, IL-10 detectability was strongly associated with histologic status (OR 102.0; 95% CI 11.4–913.9). In a reduced multivariable model adjusted for age, sex, and CRP, IL-10 detectability remained independently associated with histologic mucosal healing (adjusted OR 74.9; 95% CI 8.0–700.1; *p* = 0.00015).

The magnitude of these estimates reflects near-complete separation between groups and should be interpreted as indicative of a strong directional association rather than a precise effect size.

Continuous IL-10 analyses were therefore considered exploratory and supportive, given the high degree of left-censoring.

Discriminatory performance of IL-10 models is presented in Section 3.6. The consistency of findings across detectability-based and continuous exploratory approaches supports the robustness of the observed association, while reinforcing detectability as the primary clinically interpretable signal.

### 3.6. IL-23 Detectability and Histologic Severity

A large proportion of IL-23 measurements fell below the assay limit of detection (16.3 pg/mL). Within the IBD cohort, 53 of 59 patients (89.8%) had IL-23 values below the detection threshold.

Detectable IL-23 (≥16.3 pg/mL) was observed in 6 of 35 patients (17.1%) with active histologic inflammation and in none of the patients with histologic healing or healthy controls.

**Figure 3:**
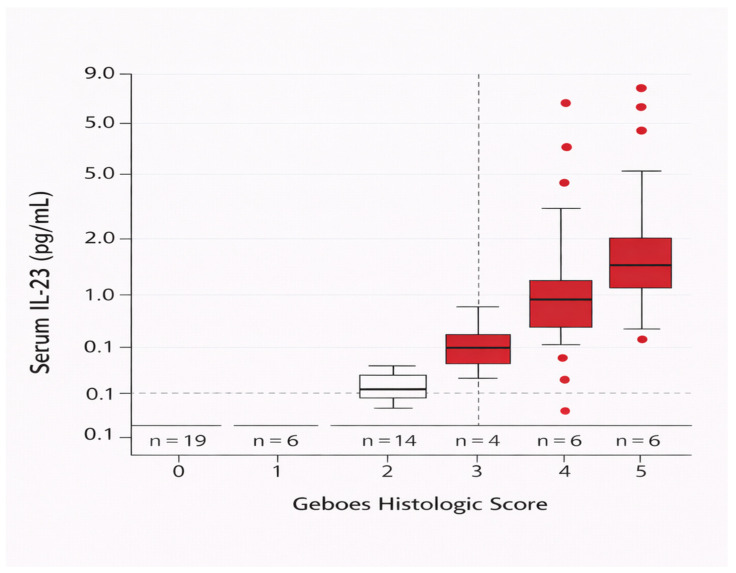
Serum IL-23 concentrations across histologic severity (Geboes score) within the IBD cohort. Detectable IL-23 values were confined to patients with moderate-to-severe microscopic inflammation (Geboes ≥ 3.1), while no detectable IL-23 values were observed in patients with histologic mucosal healing, consistent with a threshold-like pattern based on a small number of detectable cases. Boxes represent the interquartile range (IQR), with the median indicated by the central line and whiskers representing the distribution according to the Tukey method. IL-23 detectability was defined based on the assay limit of detection (LOD = 16.3 pg/mL).

Ordinal categorization of IL-23 concentrations (<LOD; detectable below the lower limit of quantification; ≥LLOQ) demonstrated a statistically detectable pattern across histologic groups (Monte Carlo exact test, *p* = 0.006; Table 6, Figure 3), although this observation is based on a very small number of detectable cases. Notably, all detectable IL-23 values were confined to patients with active histologic inflammation.

Within the IBD cohort, IL-23 detectability was confined to patients with Geboes scores ≥ 3.1, corresponding to moderate-to-severe microscopic inflammation. No patient with Geboes < 3.1 had detectable IL-23 (Fisher’s exact *p* = 0.0338).

All detectable IL-23 values occurred in patients with Geboes ≥ 3.1 (6/6), whereas none were observed in those with lower histologic scores (0/25).

In direct comparisons of histologic healing versus active inflammation, IL-23 detectability did not demonstrate graded separation across disease states, consistent with its low prevalence outside more advanced histologic activity. These findings support a pattern of severity-dependent emergence of circulating IL-23 rather than a linear association across the full spectrum of microscopic inflammation. Given the limited number of detectable observations, these results should be interpreted as descriptive and hypothesis-generating rather than as evidence of independent clinical utility.

### 3.7. Discriminatory Performance

**Figure 4:**
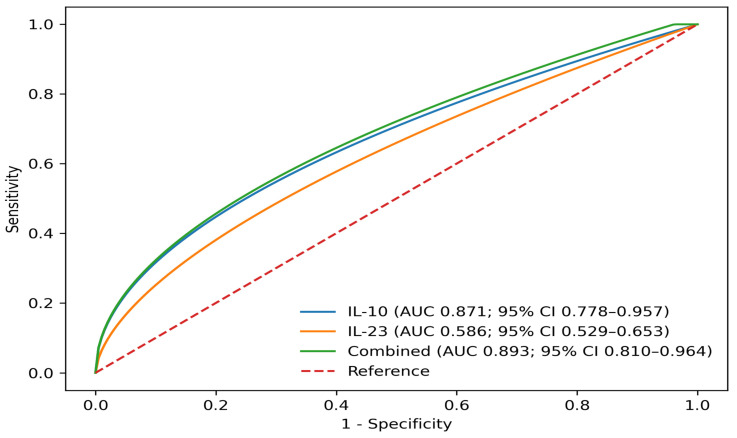
Receiver operating characteristic (ROC) curves for cytokine-based discrimination of histologic status within the IBD cohort (n = 59). Continuous IL-10 (log-transformed and LOD-adjusted), evaluated as an exploratory representation, demonstrated separation between histologic groups within this dataset (AUC 0.871; 95% CI 0.778–0.957). For IL-23, ROC analysis is presented using non-detectability (<16.3 pg/mL) as the healing-favoring direction (AUC 0.586; 95% CI 0.529–0.653), consistent with its limited graded discrimination across disease states. The combined model incorporating IL-10 (log-transformed) and IL-23 detectability (≥16.3 pg/mL) yielded a numerically higher AUC (0.893; 95% CI 0.810–0.964) without meaningful incremental improvement over IL-10 alone in this dataset. The diagonal reference line represents no-discrimination performance (AUC = 0.5). Given the high proportion of values below the assay detection limit, ROC estimates derived from continuous IL-10 should be interpreted as reflecting within-sample separation rather than stable quantitative discrimination.

Receiver operating characteristic (ROC) analyses for discrimination of histologic mucosal healing (Geboes < 2) within the IBD cohort are shown in Figure 4, with performance metrics summarized in Table 7. These analyses were performed as a secondary descriptive assessment of cytokine behavior within the cohort.

Using the Youden index, the optimal IL-10 threshold within this dataset corresponded to 4.0 pg/mL (sensitivity 75.0%, specificity 100.0%). The combined model did not significantly improve separation between histologic groups compared with IL-10 alone (DeLong *p* = 0.055). Internal validation using bootstrap resampling (2000 iterations) indicated stable performance estimates across models.

### 3.8. Internal Validation and Calibration

In addition to discrimination, calibration characteristics of the multivariable model were evaluated. The apparent AUC was 0.954, with minimal optimism observed following bootstrap correction (optimism-corrected AUC 0.928). The Brier score was 0.093, indicating good overall model fit within the dataset.

**Figure 5:**
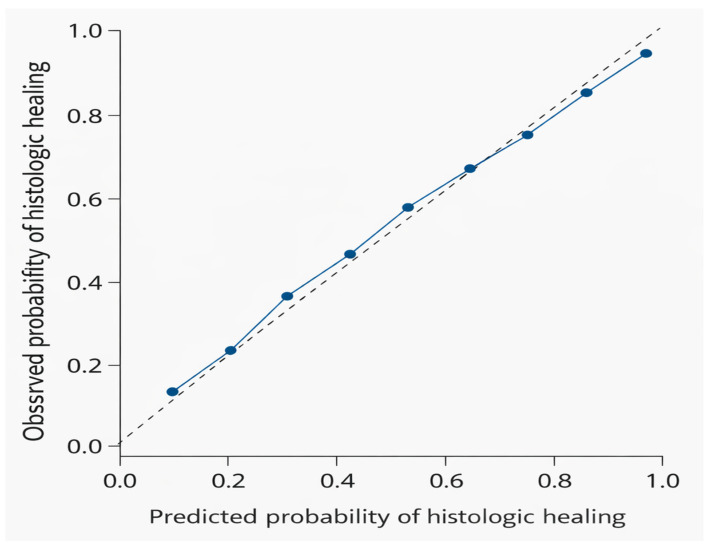
Calibration plot of predicted versus observed probability of histologic status for the multivariable model within the IBD cohort. Points represent deciles of predicted probability. The diagonal line indicates ideal agreement between predicted and observed outcomes.

Calibration analysis demonstrated a slope of 1.62 and an intercept of 0.16, suggesting acceptable agreement between predicted and observed outcomes, without evidence of substantial overfitting. The slope >1 may reflect shrinkage effects related to penalization (Table 8; Figure 5).

## 4. Discussion

In this prospective clinical cohort, circulating IL-10 and IL-23 exhibited distinct systemic associations with histologic mucosal status in inflammatory bowel disease. IL-10 demonstrated a detectability-driven inverse association with microscopic inflammation, whereas IL-23 detectability was confined to moderate-to-severe disease without graded discrimination across histologic states [20,21,22]. Together, these findings suggest complementary cytokine patterns reflecting different dimensions of microscopic inflammation.

Previous studies have examined circulating cytokines in inflammatory bowel disease, including investigations of IL-10 and related regulatory pathways. For example, prior work has reported altered IL-10 signaling and circulating cytokine patterns in association with disease activity and immune dysregulation in IBD [23,24,25,26,27,28]. However, most available studies have focused on clinical or endoscopic activity rather than histologic mucosal status, and few have evaluated systemic cytokine patterns specifically in relation to microscopic healing. In this context, the present study extends existing literature by characterizing circulating cytokine behavior across the spectrum of histologic disease activity.

A key observation was the near absence of detectable IL-10 in patients with active histologic inflammation, indicating a threshold-like separation between histologic states rather than a fully continuous quantitative gradient. Although an inverse correlation with continuous Geboes score was observed, this should be interpreted as a directional association arising from detectability differences rather than as evidence of a precise dose–response relationship. In addition, IL-10 concentrations demonstrated apparent separation across clinical groups; however, this pattern is primarily driven by clustering at the assay detection threshold in active disease. These findings suggest that circulating IL-10 may reflect aspects of systemic regulatory immune tone rather than direct quantitative secretion from inflamed tissue. Given that IL-10 is produced by multiple regulatory immune populations—including regulatory T cells, macrophages, and tolerogenic dendritic cells—and functions to constrain antigen-presenting cell activation and downstream effector cytokine amplification, reduced circulating IL-10 detectability in active microscopic inflammation may be compatible with diminished systemic regulatory containment, although this interpretation remains speculative within the cross-sectional design [29,30].

Alternatively, reduced circulating IL-10 concentrations may also reflect assay-related sensitivity limitations or compartmentalization of cytokine production within the intestinal mucosa, reinforcing that circulating measurements should be interpreted as detectability-based signals rather than precise quantitative surrogates of mucosal activity [31,32]. Accordingly, these findings should not be interpreted as supporting the use of IL-10 as a quantitative clinical biomarker in its current form, but rather as a detectability-based signal requiring further validation.

The magnitude of the observed odds ratios should be interpreted with caution. The dataset demonstrated near-complete separation between histologic groups, as the vast majority of patients with active histologic inflammation had IL-10 concentrations below the assay detection limit. Under such conditions, even penalized estimation may yield large point estimates accompanied by wide confidence intervals. Consequently, the reported odds ratios should not be interpreted as reliable quantitative estimates of effect magnitude, but rather as indicators of the direction of association under conditions of near-complete separation.

An additional methodological consideration relates to the handling of IL-10 values below the assay detection limit. Because a large proportion of measurements in the active histologic inflammation group fell below the lower limit of detection, these values were imputed as LOD/2 according to the prespecified analytical strategy. Although sensitivity analyses using detectability-based modeling yielded directionally consistent results, the possibility that LOD-based imputation influenced the magnitude of statistical associations cannot be fully excluded [33,34,35].

The high proportion of values below the assay detection limit, particularly for IL-23 and to a lesser extent IL-10, represents an important methodological constraint. In contrast to IL-10, circulating IL-23 did not demonstrate a graded association across the full histologic spectrum [36,37,38,39]. Detectable IL-23 was observed exclusively in patients with moderate-to-severe microscopic inflammation and was absent in histologic healing and in controls. All detectable cases occurred in individuals with Geboes ≥ 3.1, whereas none were observed below this threshold [40,41,42,43]. This pattern supports a severity-dependent threshold signal rather than a continuous biomarker across disease states. From an immunologic perspective, this may be compatible with IL-23 acting more as an amplifier of established inflammatory circuits than as a sensitive marker of early or residual microscopic activity.

The interpretation of IL-23 findings should be made in the context of the limited number of detectable cases. In the present cohort, IL-23 concentrations were below the limit of detection in the majority of patients, with only a small subset demonstrating detectable values. Consequently, IL-23 was modeled as a binary variable (detectable versus non-detectable), representing a pragmatic approach for heavily left-censored data. Importantly, the present dataset is not sufficient to establish the independent clinical or predictive value of IL-23, and findings should be interpreted as descriptive within this cohort.

Addition of IL-23 detectability to IL-10 in combined modeling resulted in a numerically higher AUC, with borderline statistical comparison (DeLong *p* = 0.055). However, this difference did not reach statistical significance, and therefore the combined model should not be interpreted as providing a statistically significant improvement in discriminatory performance over IL-10 alone within this dataset. Accordingly, IL-23 should not be interpreted as providing incremental predictive value beyond IL-10 in the present analysis [44,45,46,47]. Within this dataset, IL-10 appeared to account for most of the separation between histologic states, whereas IL-23 reflected a higher inflammatory threshold rather than additive graded discrimination and should be interpreted as a threshold-dependent signal rather than a graded biomarker [48,49].

From a clinical perspective, histologic mucosal healing is increasingly recognized as a meaningful therapeutic endpoint, yet its assessment remains invasive and resource-intensive. In the current clinical context, fecal calprotectin remains an established non-invasive biomarker for assessing intestinal inflammation, although it does not consistently discriminate histologic healing from residual microscopic activity. In this regard, circulating cytokines such as IL-10 and IL-23 should be considered as complementary rather than competing biomarkers. The present findings do not demonstrate superiority over existing tools but instead suggest that multidimensional immune profiling may provide additional biological insight into the spectrum of histologic disease activity. Direct comparative and longitudinal studies will be required to determine their potential additive clinical value. The association between circulating IL-10 and histologic status observed here supports further investigation as a potential correlate of microscopic disease activity [50]. These findings primarily support a mechanistic and associative interpretation rather than immediate diagnostic or prognostic application, given the cross-sectional design and lack of longitudinal validation.

A key consideration for clinical interpretation is the relationship of the present findings to established non-invasive biomarkers, particularly fecal calprotectin. In this context, the cytokine patterns observed in the present study may be viewed as complementary rather than competing biomarkers. Circulating IL-10 appeared to reflect a detectability-based dimension of systemic regulatory immune activity, whereas IL-23 detectability was associated with a higher inflammatory threshold. These features differ conceptually from fecal calprotectin, which represents a downstream marker of mucosal inflammation rather than upstream immune regulation.

Importantly, the present findings do not establish that circulating cytokines outperform or replace existing biomarkers. Rather, they suggest that multidimensional biomarker approaches integrating inflammatory and regulatory signals may provide additional insight into disease activity beyond conventional tools. However, direct comparative studies incorporating fecal calprotectin and other established markers will be required to determine the incremental clinical value of cytokine-based assessments.

The healthy control group was not included in regression modeling because the primary analytical objective focused on within-cohort associations between circulating cytokines and histologic status among patients with IBD. Inclusion of controls in regression analyses would have altered the outcome structure, effectively shifting the model toward discrimination between IBD and non-IBD states rather than between histologic healing and active microscopic inflammation. Therefore, healthy controls were used as an external reference to contextualize cytokine distributions across disease states, while regression analyses were restricted to the IBD cohort to preserve alignment with the primary biological question.

Importantly, the present findings derive from a cross-sectional analysis and therefore demonstrate associations only.

Sensitivity analyses excluding untreated patients did not materially alter the association between IL-10 and histologic status, suggesting that the findings were broadly consistent across treatment strata [51,52,53]. Moreover, circulating IL-10 concentrations did not significantly differ between biologic and non-biologic treatment groups, suggesting that the observed association was not solely driven by therapeutic exposure.

Nevertheless, the potential influence of drug-specific mechanisms of action on circulating cytokine levels should be considered. Treatment exposure was explored descriptively and through sensitivity analyses; however, it was not included as a covariate in multivariable models due to the limited events-per-variable ratio and the associated risk of model overfitting and instability. Different therapeutic classes, including biologic agents and immunomodulators, may differentially modulate cytokine pathways, which could affect circulating IL-10 and IL-23 concentrations. Although no clear treatment-related differences were observed within this dataset, the relatively limited sample size and heterogeneity of treatment regimens may have reduced the ability to detect such effects. Therefore, residual confounding related to treatment-specific immunomodulation cannot be fully excluded.

Internal validation suggested reasonably stable model behavior with limited optimism after bootstrap correction. Calibration characteristics were acceptable within the present cohort. Although derived from a single prospective cohort, the present analysis addresses a distinct biological question focusing on systemic cytokine patterns in relation to histologic disease spectrum rather than previously reported clinical or endoscopic endpoints. The absence of an independent external validation cohort represents an important limitation, as the generalizability and potential clinical applicability of these findings cannot be established within a single dataset. Accordingly, the observed associations should be interpreted as cohort-specific and require confirmation in independent populations before any broader inference can be made. Taken together, these methodological constraints—including limited sample size, high proportion of values below the limit of detection, and potential model instability—should be considered jointly, as they may collectively influence the robustness and generalizability of the observed associations. In particular, the high degree of left-censoring for IL-10 and IL-23 constrains quantitative interpretation, supporting a detectability-based and threshold-oriented framework rather than continuous biomarker inference. Similarly, the limited number of detectable IL-23 observations restricts the ability to draw conclusions regarding its independent clinical or predictive value.

### Strengths and Limitations

This study has several methodological strengths, although these should be interpreted in the context of its exploratory design and sample-size constraints. The study employed prospective enrollment with consecutive sampling, blinded histologic assessment using standardized Geboes scoring, and uniform pre-analytical handling of serum samples. Cytokine quantification was performed in duplicate under controlled laboratory conditions, and specimens were stored at −80 °C for limited and comparable durations across groups (median approximately three months), minimizing the likelihood of differential degradation bias. Statistical analyses incorporated detection-limit–aware modeling, Firth-penalized regression to address quasi-complete separation, and bootstrap resampling to assess model stability.

Several limitations warrant consideration. In addition, the overall sample size was relatively small, which may limit statistical power for detecting moderate associations and reduce the precision of effect estimates. First, given the cross-sectional design, causal inference and temporal relationships between circulating cytokines and histologic change cannot be established. Second, the single-center design and recruitment from a tertiary referral military hospital may limit external validity, and validation in independent multi-center cohorts with broader treatment distributions and disease phenotypes will be required to determine generalizability. No independent external validation cohort was available.

Serum cytokine concentrations may not fully reflect compartmentalized mucosal immune dynamics. In addition, circulating IL-10 should not be interpreted as a precise quantitative biomarker in this dataset, as a substantial proportion of values—particularly in active histologic inflammation—fell below the assay limit of detection, resulting in near-complete non-detectability in this subgroup.

Under these conditions, IL-10 is more appropriately interpreted as a detectability-based or threshold-like signal rather than a fully continuous variable, and statistical associations derived from continuous modeling should be interpreted as directional rather than quantitative. Accordingly, the primary interpretation of IL-10 in this study is based on detectability status rather than LOD-imputed continuous values.

The absence of systematic assessment of established non-invasive biomarkers, including fecal calprotectin, as well as additional cytokines involved in IBD pathogenesis (such as TNF-α, IL-6, IL-12, and IFN-γ), precluded direct comparison with established inflammatory markers and broader immune profiling approaches, limiting the ability to fully contextualize these findings within current clinical and biological frameworks.

The number of histologic healing events (n = 24) relative to the number of predictors included in multivariable modeling resulted in a modest events-per-variable ratio (approximately 3–4 events per predictor). To mitigate potential small-sample bias and quasi-complete separation, Firth-penalized logistic regression was prespecified. Nevertheless, residual model instability and the possibility of overfitting cannot be fully excluded, and effect estimates should therefore be interpreted with appropriate caution. In this context, model-derived performance metrics, including discrimination estimates, should be interpreted with particular caution, as they may reflect within-sample separation rather than stable predictive performance. Accordingly, these findings should be considered descriptive and hypothesis-generating rather than indicative of clinically applicable predictive accuracy.

A substantial proportion of cytokine measurements fell below the assay limit of detection, particularly for IL-23, resulting in partially censored biomarker distributions. Although pragmatic LOD-based imputation and detectability-based analyses were applied to address left-censored data, such approaches may influence variance structure and regression estimates. In particular, high degrees of left-censoring may introduce clustering at the detection threshold, limiting the ability to infer continuous biological gradients.

For IL-23 specifically, the very small number of detectable observations further limits statistical power and precludes reliable assessment of independent associations. Accordingly, IL-23 findings should be interpreted as descriptive and threshold-based within this cohort rather than as evidence of independent clinical or predictive utility.

Accordingly, the present findings should be interpreted as exploratory and hypothesis-generating, reflecting within-cohort patterns rather than established clinical biomarkers. Confirmation in larger independent cohorts will be required to determine the robustness and generalizability of these observations.

## 5. Conclusions

Circulating IL-10 and IL-23 exhibited distinct systemic associations with histologic mucosal status in inflammatory bowel disease. Reduced IL-10 detectability was associated with active microscopic inflammation, indicating a threshold-like separation between histologic states rather than a fully graded quantitative relationship within this cohort. In contrast, detectable IL-23 was observed only in a subset of patients with more advanced microscopic inflammation, consistent with a threshold pattern rather than a graded association and limited by the small number of detectable observations.

These findings provide exploratory evidence that circulating cytokine profiles may reflect complementary dimensions of microscopic disease activity, with IL-10 reflecting a detectability-based regulatory signal and IL-23 representing a severity-dependent inflammatory threshold rather than an independently validated biomarker within this dataset.

However, given the cross-sectional design, limited sample size, and the high proportion of values below the assay detection limit, these observations should be interpreted as hypothesis-generating. Further validation in larger independent cohorts and longitudinal studies will be required to determine the robustness, generalizability, and potential clinical relevance of these findings.

These observations may inform future biomarker development strategies by supporting the investigation of composite or multidimensional immune signatures that capture both regulatory and inflammatory axes of disease activity. In particular, approaches that integrate detectability-based and threshold-driven biomarker signals may provide improved characterization of heterogeneous inflammatory states.

These findings should be considered exploratory and cohort-specific, requiring validation in independent external populations before any clinical application and should not be interpreted as establishing independent clinical utility for IL-23 in its current form.

## Figures and Tables

**Table 1:** Baseline clinical characteristics of the IBD cohort according to histologic status.

Variable	Controls (n = 36)	IBD—Histologic Healing (n = 24)	IBD—Active Histology (n = 35)	*p*-Value
Age (years)	54 (50–60)	49 (36–56)	51 (35–62)	0.18
Male sex, n (%)	22 (61.1%)	18 (75.0%)	18 (51.4%)	0.21
Disease duration (years)	N/A	4 (1–10)	8 (3–13)	-
CRP (mg/L)	0.1 (0.1–1.6)	0.1 (0.1–0.8)	5.1 (0.8–13.0)	<0.001

Footnote: Values are presented as median (interquartile range) or n (%). Continuous variables were compared using the Kruskal–Wallis test, and categorical variables using the chi-square test or Fisher’s exact test, as appropriate. CRP: C-reactive protein.

**Table 2:** Disease characteristics according to histologic status within the IBD cohort (n = 59).

Variable	Histologic Healing (n = 24)	Active Histology (n = 35)	*p*-Value
IBD subtype, n (%)			0.558
Ulcerative colitis (UC)	19 (79.2%)	25 (71.4%)	
Crohn’s disease (CD)	5 (20.8%)	10 (28.6%)	
Disease duration (years)	5.0 (2.0–7.0)	6.0 (2.0–10.0)	0.385
Mayo endoscopic score ^†^	0.0 (0–0)	1.0 (0–2.0)	0.0007
SES-CD ^‡^	0.0 (0–0)	8.5 (7.0–10.5)	0.0072

Values are presented as median (interquartile range) or n (%). IBD subtype was compared using Fisher’s exact test; continuous variables were compared using the Mann–Whitney U test. ^†^ Mayo endoscopic score analyzed in ulcerative colitis patients only (healing n = 19; active n = 25). ^‡^ SES-CD analyzed in Crohn’s disease patients only (healing n = 5; active n = 10). SES-CD: Simple Endoscopic Score for Crohn’s Disease.

**Table 3:** Treatment exposure according to histologic status within the IBD cohort (n = 59).

Treatment Category	Histologic Healing (n = 24)	Active Histology (n = 35)	*p*-Value
None	0 (0.0%)	7 (20.0%)	
5-ASA	13 (54.2%)	10 (28.6%)	
Advanced biologic therapy	10 (41.7%)	15 (42.9%)	
Combination (biologic + azathioprine)	1 (4.2%)	3 (8.6%)	
Overall comparison			0.0549 ^†^

Values are presented as n (%). Percentages are calculated within histologic groups. ^†^ Overall comparison performed using Fisher’s exact test with Monte Carlo simulation; effect size Cramér’s V = 0.359.

**Table 4:** Serum IL-10 concentrations according to histologic status.

Group	n	IL-10 (pg/mL), Median (IQR)
Controls	36	12.1 (8.8–18.7)
IBD—Histologic Healing	24	4.5 (3.5–5.4)
IBD—Active Histology	35	1.95 (1.95–1.95)

Overall comparison: Kruskal–Wallis H = 81.454, *p* < 0.0001. Values are presented as median (interquartile range). Group differences were assessed using the Kruskal–Wallis test with pairwise Mann–Whitney U tests and Holm correction. LOD: limit of detection (3.9 pg/mL). Values below the LOD were imputed as LOD/2 (1.95 pg/mL), resulting in clustering at the lower bound in groups with a high proportion of non-detectable measurements.

**Table 5:** Multivariable association with histologic mucosal healing in the IBD cohort (n = 59).

Variable	Adjusted OR	95% CI	*p*-Value
IL-10 (log-transformed)	47.25	5.79–385.46	0.00032
IL-23 detectable (≥16.3 pg/mL)	0.52	0.06–4.57	0.555
Age (per year)	0.99	0.94–1.04	0.590
Male sex	2.49	0.58–10.69	0.219
CRP (per mg/L)	0.93	0.79–1.09	0.368
5-ASA (vs. biologic)	3.84	0.48–30.79	0.205
Combination (vs. biologic)	0.59	0.03–10.03	0.715
None (vs. biologic)	1.10	0.06–19.03	0.949

Outcome: histologic mucosal healing (Geboes score < 2). Multivariable logistic regression was performed using Firth penalized likelihood to address small-sample bias and quasi-complete separation. Reference categories were biologic therapy (treatment), female sex, and IL-23 non-detectable. IL-10 was modeled as a log-transformed continuous variable with LOD-aware imputation (values < 3.9 pg/mL imputed as 1.95 pg/mL) for exploratory purposes; given the high proportion of values below the detection limit, primary interpretation is based on detectability analyses. OR: odds ratio; CI: confidence interval.

**Table 6:** Ordinal distribution of IL-23 concentrations across histologic groups (n = 95).

Histologic Group	<LOD (<16.3 pg/mL)	Detectable < LLOQ (16.3–38.9 pg/mL)	≥LLOQ (≥39 pg/mL)
Controls (n = 36)	36 (100%)	0 (0%)	0 (0%)
IBD—Healing (n = 24)	24 (100%)	0 (0%)	0 (0%)
IBD—Active (n = 35)	29 (82.9%)	4 (11.4%)	2 (5.7%)

Overall comparison: Monte Carlo exact test (3 × 3 contingency), *p* = 0.006. Footnotes: Values are presented as number (percentage within group). LOD: limit of detection (16.3 pg/mL). LLOQ: lower limit of quantification (39 pg/mL). Detectable IL-23 values were confined to patients with active histologic inflammation, although based on a limited number of detectable observations.

**Table 7:** ROC-based descriptive performance of cytokine models in relation to histologic status (IBD cohort, n = 59).

Model	AUC (95% CI)	Optimal Cut-Off	Sensitivity (%)	Specificity (%)	PPV (%)	NPV (%)
IL-10 (continuous, log-transformed)	0.871 (0.778–0.957)	log IL-10 ≥ 1.386 (≈4.0 pg/mL)	75.0	100.0	100.0	85.4
IL-10 detectable (≥3.9 pg/mL)	0.861 (0.767–0.945)	≥3.9 pg/mL	75.0	97.1	94.7	85.0
IL-23 non-detectable (<16.3 pg/mL)	0.586 (0.529–0.653)	<16.3 pg/mL	100.0	17.1	45.3	100.0
Combined model (IL-10 + IL-23)	0.893 (0.810–0.964)	p (healing) ≥ 0.670	75.0	100.0	100.0	85.4

Footnote: Higher predicted scores correspond to greater probability of histologic mucosal healing (Geboes score < 2). Optimal cut-off values were determined using the Youden index. AUC confidence intervals were calculated using DeLong’s method, and DeLong’s test was used to compare AUCs between IL-10 alone and the combined model. PPV: positive predictive value; NPV: negative predictive value; LOD: limit of detection. The combined model was derived from logistic regression including log-transformed IL-10 and IL-23 detectability.

**Table 8:** Internal Validation and Calibration Metrics of the Multivariable Model.

Metric	Value
Apparent AUC	0.954
Optimism-corrected AUC (bootstrap, 2000)	0.928
Brier score	0.093
Calibration slope	1.62
Calibration intercept	0.16

Footnote: AUC: area under the receiver operating characteristic curve. Optimism-corrected AUC was estimated using bootstrap resampling (2000 iterations). Calibration slope and intercept were derived from logistic regression of observed outcomes on the logit of predicted probabilities. Ideal calibration corresponds to slope = 1 and intercept = 0. The Brier score reflects overall model fit within the dataset, with lower values indicating better agreement between predicted and observed outcomes.

## Data Availability

The datasets generated during the current study are available from the corresponding author on reasonable request. Data are not publicly available due to institutional and ethical restrictions.

## Abbreviations

The following abbreviations are used in this manuscript:

IBD	Inflammatory bowel disease
UC	Ulcerative colitis
CD	Crohn’s disease
IL-10	Interleukin-10
IL-23	Interleukin-23
CRP	C-reactive protein
SES-CD	Simple Endoscopic Score for Crohn’s Disease
ROC	Receiver operating characteristic
AUC	Area under the curve
LOD	Limit of detection
LLOQ	Lower limit of quantification
OR	Odds ratio
CI	Confidence interval
EPV	Events per variable
IQR	Interquartile range
4PL	Four-parameter logistic

## References

[1] Villanacci V., Antonelli E., Geboes K., Casella G., Bassotti G. (2013). Histological healing in inflammatory bowel disease: A still unfulfilled promise. World J. Gastroenterol..

[2] Turner D., Ricciuto A., Lewis A., D’amico F., Dhaliwal J., Griffiths A.M., Bettenworth D., Sandborn W.J., Sands B.E., Reinisch W. (2021). STRIDE-II: An update on the Selecting Therapeutic Targets in Inflammatory Bowel Disease (STRIDE) initiative of the IOIBD. Gastroenterology.

[3] Soubières A.A., Poullis A. (2016). Emerging biomarkers for the diagnosis and monitoring of inflammatory bowel diseases. Inflamm. Bowel Dis..

[4] Lichtenstein G.R., McGovern D.P.B. (2016). Using markers in IBD to predict disease and treatment outcomes: Rationale and a review of current status. Am. J. Gastroenterol. Suppl..

[5] Duerr R.H., Taylor K.D., Brant S.R., Rioux J.D., Silverberg M.S., Daly M.J., Steinhart A.H., Abraham C., Regueiro M., Griffiths A. (2006). A genome-wide association study identifies IL23R as an inflammatory bowel disease gene. Science.

[6] Oppmann B., Lesley R., Blom B., Timans J.C., Xu Y., Hunte B., Vega F., Yu N., Wang J., Singh K. (2000). Novel p19 protein engages IL-12p40 to form a cytokine, IL-23. Immunity.

[7] Ahern P.P., Schiering C., Buonocore S., McGeachy M.J., Cua D.J., Maloy K.J., Powrie F. (2010). Interleukin-23 drives intestinal inflammation through direct activity on T cells. Immunity.

[8] McGeachy M.J., Cua D.J., Gaffen S.L. (2019). The IL-17 family of cytokines in health and disease. Immunity.

[9] Stockinger B., Omenetti S. (2017). The dichotomous nature of T helper 17 cells. Nat. Rev. Immunol..

[10] Moschen A.R., Tilg H., Raine T. (2019). IL-12, IL-23 and IL-17 in IBD: Immunobiology and therapeutic targeting. Nat. Rev. Gastroenterol. Hepatol..

[11] Verstockt B., Salas A., Sands B.E., Abraham C., Leibovitzh H., Neurath M.F., Vande Casteele N., Ferrante M., Cleynen I., de Hertogh G. (2023). IL-12 and IL-23 pathway inhibition in inflammatory bowel disease. Nat. Rev. Gastroenterol. Hepatol..

[12] Sands B.E., Sandborn W.J., Panaccione R., O’Brien C.D., Zhang H., Johanns J., Adedokun O.J., Li K., Peyrin-Biroulet L., Van Assche G. (2019). Ustekinumab as induction and maintenance therapy for ulcerative colitis. N. Engl. J. Med..

[13] Feagan B.G., Sandborn W.J., Gasink C., Jacobstein D., Lang Y., Friedman J.R., Blank M.A., Johanns J., Gao L.-L., Miao Y. (2016). Ustekinumab as induction and maintenance therapy for Crohn’s disease. N. Engl. J. Med..

[14] Sandborn W.J., D’Haens G., Reinisch W., Panaccione R., Higgins P.D.R., Niezychowski W., Hsu Y., Zhou Q., Jiao D., Bao W. (2022). Guselkumab for the treatment of Crohn’s disease: Results from the phase 2 GALAXI study. Gastroenterology.

[15] Glocker E.-O., Kotlarz D., Boztug K., Gertz E.M., Schäffer A.A., Noyan F., Perro M., Diestelhorst J., Allroth A., Murugan D. (2009). Inflammatory bowel disease and mutations affecting the interleukin-10 receptor. N. Engl. J. Med..

[16] Kühn R., Löhler J., Rennick D., Rajewsky K., Müller W. (1993). Interleukin-10-deficient mice develop chronic enterocolitis. Cell.

[17] Mosli M.H., Zou G., Garg S.K., Feagan S.G., MacDonald J.K., Chande N., Sandborn W.J., Feagan B.G. (2015). C-reactive protein, fecal calprotectin, and stool lactoferrin for detection of endoscopic activity in IBD: Systematic review and meta-analysis. Am. J. Gastroenterol..

[18] Ouyang W., O’Garra A. (2019). IL-10 family cytokines IL-10 and IL-22: From basic science to clinical translation. Immunity.

[19] Saraiva M., O’Garra A. (2010). The regulation of IL-10 production by immune cells. Nat. Rev. Immunol..

[20] Ip W.K.E., Hoshi N., Shouval D.S., Snapper S., Medzhitov R. (2017). Anti-inflammatory effect of IL-10 mediated by metabolic reprogramming of macrophages. Science.

[21] Maaser C., Sturm A., Vavricka S.R., Kucharzik T., Fiorino G., Annese V., Calabrese E., Baumgart D.C., Bettenworth D., Borralho Nunes P. (2019). ECCO-ESGAR Guideline for Diagnostic Assessment in IBD Part 1: Initial diagnosis, monitoring of known IBD, detection of complications. J. Crohn’s Colitis.

[22] Magro F., Gionchetti P., Eliakim R., Ardizzone S., Armuzzi A., Barreiro-de Acosta M., Burisch J., Gecse K.B., Hart A.L., Hindryckx P. (2017). Third European evidence-based consensus on diagnosis and management of ulcerative colitis. Part 1: Definitions, diagnosis, extra-intestinal manifestations, pregnancy, cancer surveillance, surgery, and ileo-anal pouch disorders. J. Crohn’s Colitis.

[23] Lucaciu L.A., Ilieș M., Vesa Ș.C., Seicean R., Din S., Iuga C.A., Seicean A. (2021). Serum interleukin (IL)-23 and IL-17 profile in inflammatory bowel disease (IBD) patients could differentiate between severe and non-severe disease. J. Pers. Med..

[24] Mirsattari D., Seyyedmajidi M., Zojaji H., Haghazali M., Gooran Orimi P., Shoushtarizadeh T., Almasi S. (2012). The relation between the level of interleukin-23 with duration and severity of ulcerative colitis. Gastroenterol. Hepatol. Bed Bench.

[25] Zhu L., Shi T., Zhong C., Wang Y., Chang M., Liu X. (2017). IL-10 and IL-10 receptor mutations in very early onset inflammatory bowel disease. Gastroenterol. Res..

[26] Rastegar Hoseini R., Rahim H.A., Saifalddin D.L., Kareem D.A., Fatah A.M. (2025). Exercise intensity-mediated regulation of gut epithelial cells and immune function in gut microbiota dysbiosis. J. Transl. Med..

[27] Frigerio S., Khan H.N., Amini M., Mol B., Neefjes-Borst A., Wildenberg M.E., Ponsioen C.Y., D’Haens G.R., Vercoulen Y., Grootjans J. (2025). Spatial transcriptomics and immunophenotyping uncover chronic inflammation-induced immune adaptations favoring dysplasia development in patients at risk of colitis-associated cancer. J. Crohn’s Colitis.

[28] Godala M., Gaszyńska E., Malecka-Wojciesko E. (2025). Association between pro-inflammatory potential of diet and inflammatory parameters in a group of patients with inflammatory bowel disease. Nutrients.

[29] Danese S., Fiocchi C. (2011). Ulcerative colitis. N. Engl. J. Med..

[30] D’Haens G., Sandborn W.J., Feagan B.G., Geboes K., Hanauer S.B., Irvine E.J., Lémann M., Marteau P., Rutgeerts P., Schölmerich J. (2007). A review of activity indices and efficacy endpoints for Crohn’s disease. Gastroenterology..

[31] Martinos N., Lazaris A.C., Kroupis C., Kranidiotis G., Thomopoulou G.-E. (2026). Interleukin Signatures as Prognostic Biomarkers in Ulcerative Colitis: From Immune Pathways to Clinical Prediction. Curr. Issues Mol. Biol..

[32] Chakraborty S. (2012). IL23 as a novel serum based biomarker in ulcerative colitis—An editorial. Gastroenterol. Hepatol. Bed Bench.

[33] Kikly K., Liu L., Na S., Sedgwick J.D. (2006). IL-23/Th17 axis as therapeutic target. Curr. Opin. Immunol..

[34] Abraham C., Dulai P.S., Vermeire S., Sandborn W.J. (2017). Lessons learned from trials targeting cytokine pathways in patients with inflammatory bowel diseases. Gastroenterology.

[35] Bain C.C., Bravo-Blas A., Scott C.L., Gomez Perdiguero E., Geissmann F., Henri S., Malissen B., Osborne L.C., Artis D., Mowat A.M. (2014). Constant replenishment from circulating monocytes maintains the macrophage pool in the intestine of adult mice. Nat. Immunol..

[36] Gagliani N., Magnani C.F., Huber S., Gianolini M.E., Pala M., Licona-Limón P., Guo B., Herbert D.R., Bulfone A., Trentini F. (2013). Coexpression of CD49b and LAG-3 identifies human and mouse T regulatory type 1 cells. Nat. Med..

[37] Huber S., Gagliani N., Esplugues E., O’Connor W., Huber F.J., Chaudhry A., Kamanaka M., Kobayashi Y., Booth C.J., Rudensky A.Y. (2011). Th17 cells express interleukin-10 receptor and are controlled by Foxp3^−^ and Foxp3+ regulatory CD4+ T cells in an interleukin-10-dependent manner. Immunity.

[38] Lee Y., Awasthi A., Yosef N., Quintana F.J., Xiao S., Peters A., Wu C., Kleinewietfeld M., Kunder S., Hafler D.A. (2012). Induction and molecular signature of pathogenic Th17 cells. Nat. Immunol..

[39] Ghoreschi K., Laurence A., Yang X.-P., Tato C.M., McGeachy M.J., Konkel J.E., Ramos H.L., Wei L., Davidson T.S., Bouladoux N. (2010). Generation of pathogenic Th17 cells in the absence of TGF-β signalling. Nature.

[40] O’Shea J.J., Paul W.E. (2010). Mechanisms underlying lineage commitment and plasticity of helper CD4+ T cells. Science.

[41] Kurumi H., Yokoyama Y., Hirano T., Akita K., Hayashi Y., Kazama T., Isomoto H., Nakase H. (2024). Cytokine profile in predicting the effectiveness of advanced therapy for ulcerative colitis: A narrative review. Biomedicines.

[42] Bertani L., Caviglia G.P., Antonioli L., Pellicano R., Fagoonee S., Astegiano M., Saracco G.M., Bugianesi E., Blandizzi C., Costa F. (2020). Serum interleukin-6 and -8 as predictors of response to vedolizumab in inflammatory bowel diseases. J. Clin. Med..

[43] Neurath M.F. (2019). IL-23 in inflammatory bowel diseases and colon cancer. Cytokine Growth Factor Rev..

[44] Uchiyama K., Takagi T., Mizushima K., Asaeda K., Kajiwara-Kubota M., Kashiwagi S., Toyokawa Y., Tanaka M., Hotta Y., Naito Y. (2022). Mucosal interleukin-8 expression as a predictor of subsequent relapse in ulcerative colitis patients with Mayo endoscopic subscore 0. J. Gastroenterol. Hepatol..

[45] Rosenberg L., Nanda K.S., Zenlea T., Gifford A., Lawlor G.O., Falchuk K.R., Wolf J.L., Cheifetz A.S., Goldsmith J.D., Moss A.C. (2013). Histologic markers of inflammation in patients with ulcerative colitis in clinical remission: Correlates of histological inflammation. Clin. Gastroenterol. Hepatol..

[46] Couto M.R., Gonçalves P., Magro F., Martel F. (2020). Microbiota-derived butyrate regulates intestinal inflammation: Focus on inflammatory bowel disease. Pharmacol. Res..

[47] Mudter J., Neurath M.F. (2007). IL-6 signaling in inflammatory bowel disease. Inflamm. Bowel Dis..

[48] Ligumsky M., Simon P.L., Karmeli F., Rachmilewitz D. (1990). Role of interleukin 1 in inflammatory bowel disease—Enhanced production during active disease. Gut.

[49] Daig R., Andus T., Aschenbrenner E., Falk W., Schölmerich J., Gross V. (1996). Increased interleukin-8 expression in ulcerative colitis. Gut.

[50] Mazzucchelli L., Hauser C., Zgraggen K., Wagner H., Hess M., Laissue J.A., Mueller C. (1994). Expression of interleukin-8 gene in inflammatory bowel disease is related to the histological grade of active inflammation. Am. J. Pathol..

[51] Xue M., Shi L., Wang W., Chen S., Wang L. (2018). An overview of molecular profiles in ulcerative colitis-related cancer. Inflamm. Bowel Dis..

[52] Mosli M.H., Feagan B.G., Sandborn W.J., D’Haens G., Behling C., Kaplan K., Driman D.K., Shackelton L.M., Baker K.A., Macdonald J.K. (2014). Histologic evaluation of ulcerative colitis: A systematic review of disease activity indices. Inflamm. Bowel Dis..

[53] Marchal-Bressenot A., Salleron J., Boulagnon-Rombi C., Bastien C., Cahn V., Cadiot G., Diebold M.-D., Danese S., Reinisch W., Schreiber S. (2017). Development and validation of the Nancy histological index. Gut.

